# Investigation of the human nasal microbiome in persons with long- and short-term exposure to methicillin-resistant *Staphylococcus aureus* and other bacteria from the pig farm environment

**DOI:** 10.1371/journal.pone.0232456

**Published:** 2020-04-30

**Authors:** Md Zohorul Islam, Thor Bech Johannesen, Berit Lilje, Tinna Ravnholt Urth, Anders Rhod Larsen, Øystein Angen, Jesper Larsen

**Affiliations:** 1 Department of Bacteria, Parasites, and Fungi, Statens Serum Institut, Copenhagen, Denmark; 2 Department of Veterinary and Animal Sciences, Faculty of Health and Medical Sciences, University of Copenhagen, Frederiksberg, Denmark; 3 Department of Infectious Disease Epidemiology and Prevention, Statens Serum Institut, Copenhagen, Denmark; University of Minnesota Twin Cities, UNITED STATES

## Abstract

Since its emergence in the early 2000s, livestock-associated methicillin-resistant *Staphylococcus aureus* clonal complex 398 (LA-MRSA CC398) has led to an increasing number of human infections in Denmark and other European countries with industrial pig production. LA-MRSA CC398 is primarily associated with skin infections among pig farm workers but is also increasingly recognized as a cause of life-threatening disease among elderly and immunocompromised people. Pig farm workers may serve as vehicles for the spread of LA-MRSA CC398 and other farm-origin bacteria between farms and into the general population. Yet, little is known about the bacterial community dynamics in pig farm workers and other persons with long- and short-term exposure to the pig farm environment. To gain insight into this, we investigated the nasal microbiomes in pig farm workers during a workweek on four LA-MRSA CC398-positive pig farms, as well as in short-term visitors two hours before, immediately after, and 48 hours after a 1-hour visit to another LA-MRSA CC398-positive pig farm. *S*. *aureus* and LA-MRSA CC398 carriage was quantified by means of culture, and the composition of the bacterial communities was investigated through sequencing of the 16S rRNA gene. Pig farm workers often carried LA-MRSA CC398 and other bacteria from the pig farm environment, both at work and at home, although at lower levels at home. In contrast, short-term visitors were subject to a less dramatic and rapidly reversible change in the nasal bacterial community composition. These results suggest that pig farm workers may be an important source of LA-MRSA CC398 and perhaps other pathogens of human and veterinary relevance.

## Introduction

*Staphylococcus aureus* is an opportunistic pathogen that is carried by approximately 30–50% of the human population worldwide [1]. The anterior nares are the primary niche for *S*. *aureus*, although the throat and perineum are also important reservoirs [1]. Individuals can be assigned into persistent carriers (~20%), intermittent carriers (~30%), and non-carriers (~50%), while it has been argued that there are only two types of nasal carriers: persistent carriers and others [2,3]. Persistent nasal carriers often harbor *S*. *aureus* at higher loads and for longer periods and have an increased risk of *S*. *aureus* infection than intermittent carriers or non-carriers [2–4]. *S*. *aureus* carriage is highly dependent on the composition of the prevailing nasal bacterial community, which may reflect antagonistic or mutualistic interspecies interactions [5]. For example, the anterior nares are known to harbor bacterial species that may secrete various anti-*S*. *aureus* compounds, such as the serine protease Esp produced by *Staphylococcus epidermidis* and lugdunin, a novel thiazolidine-containing cyclic peptide antibiotic produced by *Staphylococcus lugdunensis* [6,7].

The increasing spread of methicillin-resistant *S*. *aureus* (MRSA) in healthcare settings, the community, and in food animals represents a serious challenge to global health, as these strains are resistant to a broad spectrum of clinically important β-lactam antibiotics, such as penicillins, most cephalosporins, and carbapenems [8]. In Europe, the pig industry constitutes an expanding reservoir for livestock-associated MRSA clonal complex 398 (LA-MRSA CC398) [9]. The prevalence of nasal carriage can be very high (>85%) among people who work on pig farms that are positive for LA-MRSA CC398 [10,11], whereas it remains relatively low in the general population (e.g., 0.2% in the German general population) [12]. Nonetheless, one-third of all LA-MRSA CC398 infections occur in people people with no livestock contact [13]. Furthermore, a longitudinal study from Germany showed that pig farm workers may carry LA-MRSA CC398 for extensive periods outside the farm environment, sometimes for several weeks, although it should be noted that the study design did not allow the investigators to evaluate changes in bacterial load over time [14]. As a consequence, this clone is now an important cause of mainly skin infections among farmers and their families but has also been associated with bacteremia and deaths among elderly and immunocompromised individuals living in the same areas [13,15–17]. In contrast, a recent study from Denmark showed that LA-MRSA CC398 is eliminated in a matter of hours in volunteers following brief exposure (1 h) to a positive pig farm [18].

Along with the risk of *S*. *aureus* infection, people exposed to the pig farm environment may also transport LA-MRSA CC398 to other animals and farms as well as to their households and the general population. In addition, it seems reasonable to assume that the nasal environment of these persons may also serve as a hub for the spread of other bacteria present in the pig farm environment. In this study, we sought to determine the composition and temporal dynamics of the nasal microbiome in people during and after long- and short-term exposure to LA-MRSA CC398-positive pig farms.

## Materials and methods

### Ethical statement

The use of data and nasal swabs from pig farm workers and volunteers was approved by the Danish Data Protection Agency (protocol no. 2001-14-0021) and the National Committee on Health Research Ethics (protocol no. H-15013814), respectively, which waived the need for informed consent from pig farm workers, whereas volunteers were asked to provide written informed consent data to participate in the study.

### *S*. *aureus* and MRSA carriage in pig farm workers

Participants were recruited among pig farm workers on four pig farms (Table 1) that were found positive for LA-MRSA CC398 in a survey conducted in 2014 [19]. A total of 16 pig farm workers, four from each farm, volunteered to participate in the study, which took place in July and October 2016 (Table 2). Participants were trained to collect nasal swabs by rotating a sterile FLOQSwab (ESwab Liquid Amies Collection and Transport System, Copan, Brescia, Italy) five times in both anterior nares. Swabs were collected twice daily over an entire workweek from Monday morning to the following Monday morning. The first swab was collected in the morning (i.e., before going to work) while the second was collected in the afternoon (i.e., at the end of a workday). The sampling moments for each pig farm worker are visualized in S1 Fig.

**Table 1:** Pig farms included in this study.

Pig farm	Participants	No. of sows	No. of weaners	No. of finishers
Pig farm workers				
A	1–4	1,000	1,300	0
B	5–8	800	3,000	200
C	9–12	650	2,300	5,740
D	13–16	885	3,304	2,400
Volunteers and pigs				
E	8 volunteers	0	2,400	450
	4 pigs			

**Table 2:** Study population.

Study population	No.	Gender	Median age, years (range)	Health status	Antibiotic therapy	Use of face mask
Pig farm workers	16	2 women	32.5 (21–54)	Healthy (*n* = 16)	No (*n* = 16)	No (*n* = 16)
		14 men			Yes (*n* = 0)	Yes (*n* = 0)
Volunteers	8	7 women	25.5 (22–44)	Healthy (*n* = 8)	No (*n* = 8)	No (*n* = 8)
		1 man			Yes (*n* = 0)	Yes (*n* = 0)

Swabs were stored in 1 ml of liquid Amies medium at 4°C and transported to the laboratory within 12 hours of sample collection by a member of the study group. Questionnaire responses were collected at the end of the study period. Each participant reported demographic information, medical history, and occupational activities (e.g., gender, age, health status, antibiotic therapy, daily workhours, and use of face masks). For each nasal swab, an 100-μl aliquot of liquid Amies medium was used to make 10-fold dilutions in 0.1% Triton X-100 (Sigma-Aldrich, St. Louis, USA), of which 100 μl were pipetted directly onto SaSelect (Bio-Rad, Hercules, CA, USA) and Brilliance MRSA 2 (Oxoid, Basingstoke, United Kingdom) agar plates using a sterile spreader. Plates were incubated at 35°C for 24 hours and the number of CFUs were counted. If there was no growth on either plate after 24 hours, 100 μl of liquid Amies medium was inoculated into 5 ml of Mueller-Hinton broth (Oxoid, Basingstoke, United Kingdom) supplemented with 6.5% NaCl and incubated overnight at 35°C. The enrichment broth was then streaked onto SaSelect and Brilliance MRSA 2 agar plates followed by incubation at 35°C for 24 hours. From each sample, at least one presumptive *S*. *aureus* colony from each plate was archived at -80°C in ox broth supplemented with 15% glycerol (SSI Diagnostica, Hillerød, Denmark). DNA was extracted by boiling 2–3 fresh colonies from an overnight blood agar plate culture in 200 μl sterile water for 5 minutes, followed by centrifugation at 4,500 rpm at 4°C for 5 min. A multiplex PCR assay was employed to detect the *S*. *aureus*-specific *spa* gene, the CC398-specific *sau1-hsdS1* variant, as well as the *mecA*, *mecC*, *scn*, and *lukF-PV* genes [20].

Variables were compared between groups using Fisher’s exact test for categorical data, while Wilcoxon matched-pairs signed rank test was used to evaluate differences in MRSA counts in nasal swabs collected from the same individual (GraphPad Prism software, version 5, GraphPad, La Jolla, California). The significance level was set at α = 0.05.

### Microbiome analysis

For each of the 16 pig farm workers, we characterized the microbiota in a subset of four nasal swabs collected in the morning (*n* = 3) or in the afternoon (*n* = 1), resulting in a total number of 64 samples. The sampling moments for each pig farm worker are visualized in S1 Fig. For comparative purposes, we also analyzed nasal swabs from another study, which investigated *S*. *aureus* and MRSA carriage in volunteers visiting another LA-MRSA CC398-positive pig farm (Table 1) twice for one hour in 2016 (visit 1 and 2) [18]. From that study, we included samples collected from eight volunteers two hours before, immediately after, and 48 hours after each visit, resulting in a total number of 48 samples (Table 2). None of the included volunteers had contact to animal farms prior to or during the study period. In addition, we also included four nasal and four skin swabs collected from four individual pigs during the first visit [18]. Nasal swabs from volunteers were collected in the same way as for pig farm workers by rotating a sterile FLOQSwab (ESwab Liquid Amies Collection and Transport System, Copan, Brescia, Italy) five times in both anterior nares. All swabs were stored in liquid Amies medium at -80°C before DNA extraction.

DNA was isolated from the samples using the FastDNA SPIN Kit (Qbiogene, Carlsbad, CA, USA) according to the manufacturer’s instructions. The V3-V4 variable region of the 16S rRNA gene was amplified and sequenced using a previously described protocol [21]. The sequence data have been deposited in the European Nucleotide Archive under BioProject accession number PRJEB24042.

Raw sequence reads were processed and de-replicated using the “BION-meta” package (Danish Genome Institute, Denmark). In brief, pairs from the raw sequence reads were extracted using the given primer sets with both mates present. The extracted pairs were quality-trimmed with the minimum quality filter set at 99% for at least 14 of 15 bases for forward reads and at least 28 of 30 bases for reverse reads. The low-complexity filter was set to a minimum of 50 reads. Pair mates were joined into single sequences if there were overlaps of at least 18 bases with at least 90% similarity. Both overlapping and non-overlapping pairs were used for de-replication by converting identical pairs into one.

The joined paired-end reads were analyzed with a custom and streamlined workflow platform called Microbiome Helper version 1.0.1 [22]. Chimeric sequences were removed with VSEARCH [23] using the script ‘*chimera_filter*.*pl’*. Reads were clustered into operational taxonomic units (OTUs) at 97% sequence similarity using QIIME wrapper scripts [24]. The open-reference OTU picking script ‘*pick_open_reference_otus*.*py’* was used in combination with the program SortMeRNA [25] for reference picking and with the program SUMACLUST [26] for *de novo* OTU picking, with the subsampling percentage set at 10%. Low-confidence OTUs called by <0.1% of the reads were removed using the script ‘*remove_low_confidence_otus*.*py’*. The script ‘*single_rarefaction*.*py’* was used to rarefy the OTU table to a depth of 3,130 reads per sample, which was the smallest number of reads within the sample set.

Community state types (CSTs) were identified through hierarchical clustering of samples using the “vegan” package version 2.4.4 [27] in R version 3.3.3 [28] with Bray-Curtis dissimilarities and Ward’s linkage method. Hierarchical clustering of OTUs was performed with Euclidean distances using R version 3.3.3 [28]. We used the “gplots” package version 3.0.1 [29] to generate a heatmap of a subset of the most abundant (≥1% of each community) OTUs within each CST.

We calculated the within-sample alpha-diversity, measured by the Shannon diversity index, and compared Shannon diversity indices between groups with the QIIME workflow ‘*alpha_rarefaction*.*py’* using default settings.

## Results and discussion

### Prevalence and survival of *S*. *aureus* and MRSA in pig farm workers

The pig farm workers provided 221 samples of a possible 240 (S1 Fig). Of these, 87% (192/221) of the samples resulted in growth of presumptive MRSA colonies on Brilliance MRSA 2 agar. PCR analysis showed that all 221 isolates belonged to LA-MRSA CC398. The MRSA status in 76 pairs of samples collected from the same individual at the end of a workday and the following morning were compared to evaluate intranasal survival outside the farm environment. MRSA was detected in 86% (65/76) and 87% (66/76) of samples collected at the end of a workday and the following morning, respectively. The median count of MRSA in the 65 individuals who were MRSA-positive at the end of a workday was 16,000 CFUs per swab (range, 10–11,900,000) at the end of the workday and 9,800 CFUs per swab (range, 0–3,000,000) the following morning, corresponding to a median decrease of 1,000 CFUs in each individual (*P* = 0.0111 by Wilcoxon matched-pairs signed rank test). We did not attempt to evaluate changes in MRSA status and amount of MRSA in nasal swabs collected from the same individual at the end of a workday and ≥24 hours after a workday because of small sample sizes. It should be noted, however, that some pig farm workers (e.g., subjects 11, 14, and 16) carried high numbers of MRSA in samples collected up to five days after their last workday (S1 Fig). In contrast, two pig farm workers (subjects 8 and 12) were almost always negative for MRSA (S1 Fig). Samples from four pig farm workers (subjects 8, 11, 12, and 16), including the two who were mostly negative for MRSA (subjects 8 and 12), yielded much higher bacterial counts on SaSelect agar than on Brilliance MRSA 2 agar, both at work and at home, which suggests that they may harbor higher loads of methicillin-susceptible *S*. *aureus* (MSSA) than MRSA (S1 Fig). PCR analysis of 148 presumptive *S*. *aureus* isolates growing on SaSelect agar confirmed that the four pig farm workers predominantly carried MSSA (Table 3). The results furthermore showed that all 108 MSSA isolates from three of the pig farm workers (subjects 11, 12, and 16) belonged to CC398, suggesting that they originate from the pig farm environment. In contrast, all 40 MSSA isolates from the remaining pig farm worker (subject 8) did not belong to CC398, and their origin therefore remains unclear.

**Table 3:** PCR analysis of 148 presumptive *S*. *aureus* isolates from selected pig farm workers following growth on SaSelect agar.

Pig farm worker	Sampling moment	No. of presumptive *S*. *aureus* isolates	No. of *S*. *aureus* colonies	
			MSSA	MRSA
8	Monday morning	10	10	0
8	Monday afternoon	10	10	0
8	Friday afternoon	10	10	0
8	Sunday morning	10	10	0
11	Monday morning	10	10	0
11	Monday afternoon	10	10	0
11	Monday morning	10	10	0
12	Monday morning	8	8	0
12	Monday afternoon	10	10	0
12	Friday afternoon	10	10	0
12	Monday morning	10	10	0
16	Monday morning	10	8	2
16	Monday afternoon	10	8	2
16	Friday afternoon	10	10	0
16	Monday morning	10	10	0
Total		148	144	4

These results confirm that most pig farm workers carry high loads of LA-MRSA CC398 outside the farm environment, sometimes for several days as documented previously [14]. In contrast to pig farm workers, Angen et al. have shown that MRSA carriage among short-term visitors is transient [18]. In that study, 94% of the short-term visitors acquired MRSA during the visit, but the median MRSA count was only 55 CFUs in nasal swabs collected immediately after the visit, decreasing to zero CFUs in nasal swabs collected two hours later [18]. The observed differences in the duration and level of MRSA carriage are important as they are both likely to play a critical role in the transmission of LA-MRSA CC398 to humans outside the farm environment. Interestingly, two pig farm workers carried predominantly MSSA during the entire study period. Previous studies suggest that MSSA carriage may have a protective effect against MRSA acquisition in healthcare settings [30–32], and it is possible that a similar correlation exists in the pig farm environment. On the other hand, a study from the Netherlands showed that patients who carry MSSA at hospital admission are more likely to acquire exogenous *S*. *aureus* strains during their hospital stay [33].

### Nasal bacterial community composition in pigs, short-term visitors, and pig farm workers

We characterized the microbiota in 48 nasal swabs collected from eight short-term visitors two hours before, immediately after, and 48 hours after their two visits to a LA-MRSA CC398-positive pig farm as well as in four nasal and four skin swabs collected from four individual pigs in the same farm [18]. In addition, 64 nasal swabs collected from 16 pig farm workers on four different farms were included in the analysis (S1 Fig). This produced a dataset consisting of 11,273 reads per sample and a total of 3,419 unique OTUs.

**Fig 1:**
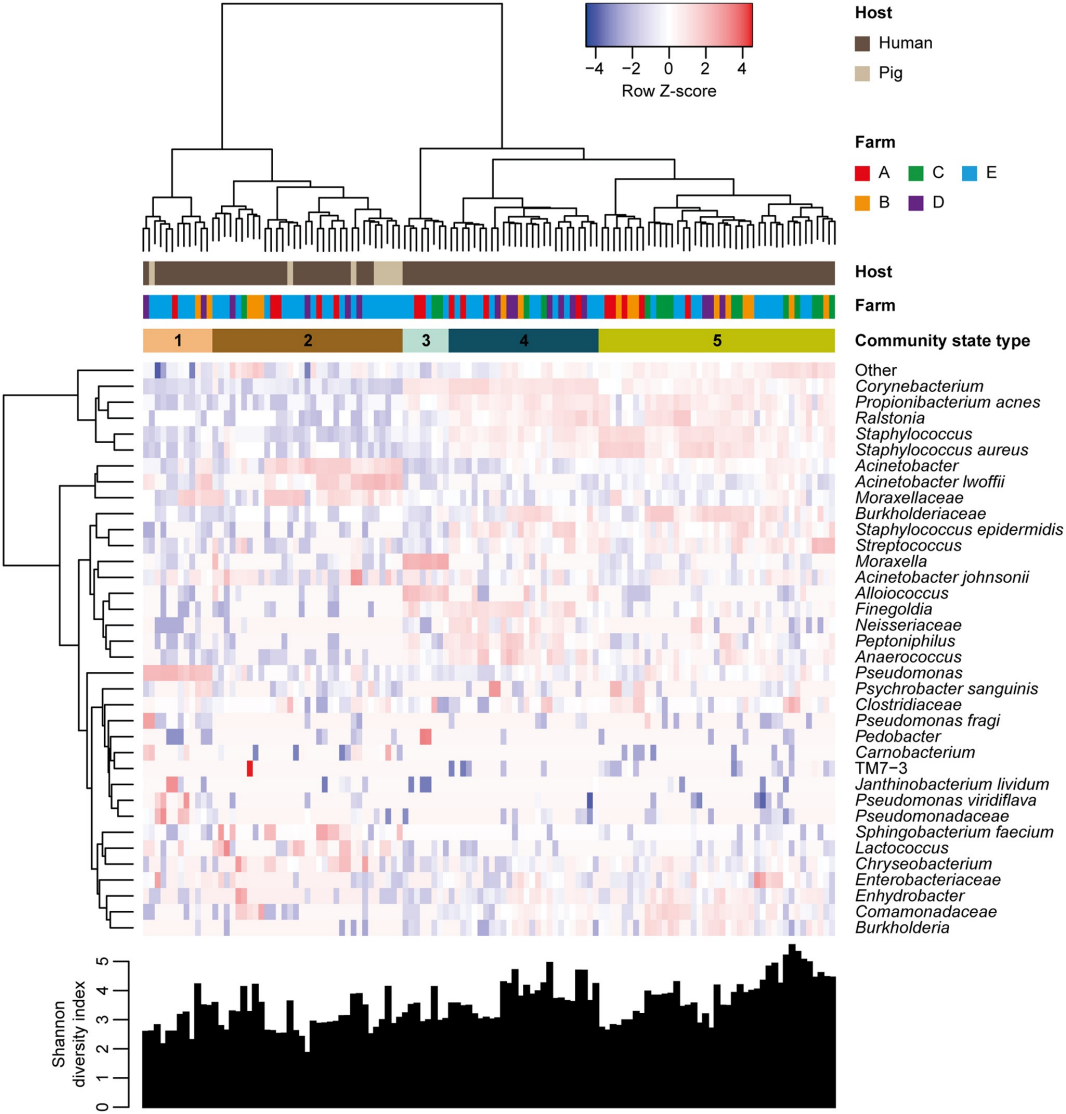
Bacterial community composition in pigs, short-term visitors, and pig farm workers. The heatmap shows log_e_-transformed proportions of operational taxon units (OTUs) found in samples from pigs, short-term visitors, and pig farm workers. Hierarchical clustering of both samples and OTUs and Shannon diversity indices calculated for each sample are shown. Row Z-score = (value—row mean)/row standard deviation.

The nasal bacterial communities were grouped according to community composition (Fig 1). The analysis revealed five major CSTs, referred to as CST1, CST2, CST3, CST4, and CST5, which contained 812, 1,788, 848, 1,733, and 2,683 OTUs, respectively. There were no significant differences between Shannon diversity indices of the communities (Fig 1). The nasal and skin bacterial communities of pigs belonged to either CST1 (*n* = 1) or CST2 (*n* = 7), which were dominated by the genera *Pseudomonas* (54% of the community) and *Acinetobacter* (37% of the community), respectively. Interestingly, CST1 and CST2 had low proportions of *S*. *aureus* (<0.5% of each community). The widespread distribution and abundance of *Pseudomonas* and *Acinetobacter*, which both belong to the order Pseudomonadales, as well as the low abundance of *S*. *aureus*, is reminiscent of previously published studies of the microbial diversity in the pig nose [34–39]. In contrast, CST3, CST4, and CST5 were dominated by the genera *Moraxella* (44% of the community), *Corynebacterium* (30% of the community), and *Staphylococcus* (40% of the community), respectively, which are known members of nasal bacterial communities present in the general human population [5,40–42].

### Exposure to the pig farm environment causes a temporary change in the nasal microbiome of short-term visitors

**Fig 2:**
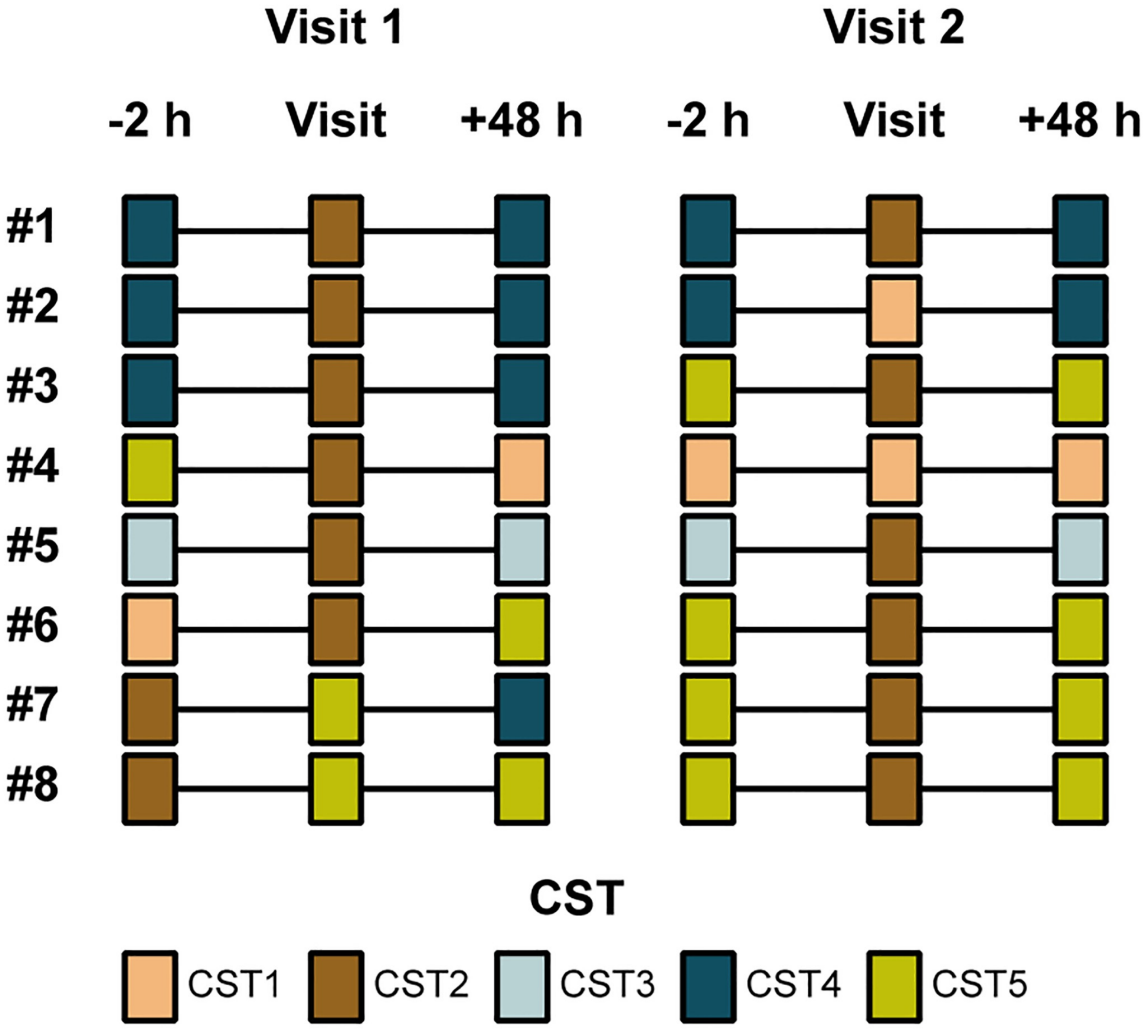
Bacterial community composition in short-term visitors. Community state types (CSTs) were determined in samples collected from volunteers two hours before, immediately after, and 48 hours after their two visits to a LA-MRSA CC398-positive pig farm.

CST1 and CST2 were present at higher frequencies in samples collected from short-term visitors immediately after their visits to the pig farm than in samples collected two hours before or 48 hours after the visits (88% [14/16] vs. 13% [2/16] vs. 25% [4/16]; *P* ≤ 0.001 by Fisher’s exact test). It follows that CST3, CST4, and CST5 were identified in most of the nasal swabs collected two hours before and 48 hours after each visit (Fig 2). Similarly, all 16 samples collected immediately after the visits were previously shown to be positive for MRSA, whereas all 32 samples collected two hours before or 48 hours after the visits were MRSA-negative [18]. Together, these findings show that brief exposure to the pig farm environment results in a rapidly reversible change in the nasal bacterial community composition.

### Pig farm workers carry farm-origin bacteria at home

The analysis of the microbiota in 64 nasal swabs collected from pig farm workers revealed highly heterogeneous patterns of variation in bacterial community composition and relative abundances of bacterial taxa between individuals and within the same individual (S1 Fig). CST1 and CST2 were present at higher frequencies in samples collected from pig farm workers at the end of a workday than in samples collected at home (43% [6/14] vs. 22% [11/50]), although the observed difference was not statistically significant by Fisher’s exact test. In addition, there were no statistically significant associations between nasal bacterial community composition at the CST level and the MRSA status in the 64 nasal swabs (*P* > 0.05 by Fisher’s exact test) (Table 4). The former finding is in line with previous studies showing that exposure to the pig farm environment affects the nasal microbiome of pig farm workers at work [38,39]. Interestingly, CST2 was present in some pig farm workers (e.g., subjects 1, 6, 14, and 16) up to four days after their last workday (S1 Fig), supporting that they may carry these bacteria for long periods and thus serve as a hub for the spread of pathogens from the pig farm environment to other animals and farms as well as to their households and the general population.

**Table 4:** LA-MRSA CC398 carriage vs. community state type among pig farm workers.

Community state type	LA-MRSA CC398 status	
	+	-
1	4	1
2	11	1
3	2	2
4	11	4
5	26	2

### Strengths and weaknesses of the study

The longitudinal study design was chosen to reduce potential biases associated with the high degree of interpersonal variability in terms of nasal bacterial community composition [43]. The study also has some limitations. First, the study included a limited number of participants and pig farms. Therefore, the results may not be generalizable to all pig farms or to other pig farm workers and visitors. Second, participants collected their own nasal swabs, which raises the possibility of contamination by airborne bacteria and bacteria located on skin surfaces surrounding the nostrils. Third, all the pig samples originated from a single farm visited by the volunteers, and we may therefore have underestimated the microbial diversity in the Danish pig production system. However, we did not identify any additional CSTs among the pig farm workers from the other pig farms, which supports that CST1 and CST2 represent the bacterial communities of all the pig farms included in this study. Fourth, the observed differences between volunteers and pig farm workers should be interpreted with caution as they may have been exposed to varying amounts of LA-MRSA CC398 and other bacteria on the different pig farms. Fifth, the original composition of the nasal bacterial communities in the pig farm workers could not be determined as they had already been exposed to MRSA and other farm-origin bacteria in the period immediately before the start of the study. Sixth, most OTUs were classified at the genus level or above, which limited our ability to access bacterial community dynamics at the species level and associations between specific bacteria and the MRSA status. Future studies should therefore aim to increase the taxonomic resolution, for example through the use of culture-based methods [44], sequencing of the full 16S rRNA gene [45], or both.

## Conclusions

The present study shows that the nasal microbiome of pig farm workers contain farm-origin bacteria, including LA-MRSA CC398, both at work and at home. In contrast, short-term visitors are subject to a less dramatic and rapidly reversible change in the nasal bacterial community composition. These results suggest that pig farm workers are more likely to serve as a vehicle for LA-MRSA CC398 and perhaps other pathogens of human and veterinary importance than short-term visitors.

## Supporting information

S1 Fig Sampling moments and results for each pig farm worker. Log_10_-transformed CFU counts of *Staphylococcus aureus* and methicillin-resistant *S*. *aureus* and distribution of community state types (CSTs) among pig farm workers are shown. The dashed line indicates the limit of CFU detection.
(TIF)

## References

[1] Lowy FD. *Staphylococcus aureus* Infections. N Engl J Med. 1998;339: 520–532. 10.1056/NEJM199808203390806 9709046

[2] Wertheim HF, Melles DC, Vos MC, van Leeuwen W, van Belkum A, Verbrugh HA, et al The role of nasal carriage in *Staphylococcus aureus* infections. Lancet Infect Dis. 2005;5: 751–762. 10.1016/S1473-3099(05)70295-4 16310147

[3] van Belkum A, Verkaik NJ, de Vogel CP, Boelens HA, Verveer J, Nouwen JL, et al Reclassification of *Staphylococcus aureus* nasal carriage types. J Infect Dis. 2009;199: 1820–1826. 10.1086/599119 19419332

[4] von Eiff C, Becker K, Machka K, Stammer H, Peters G. Nasal carriage as a source of *Staphylococcus aureus* bacteremia. N Engl J Med. 2001;344: 11–16. 10.1056/NEJM200101043440102 11136954

[5] Liu CM, Price LB, Hungate BA, Abraham AG, Larsen LA, Christensen K, et al *Staphylococcus aureus* and the ecology of the nasal microbiome. Sci Adv. 2015;1: e1400216 10.1126/sciadv.1400216 26601194 PMC4640600

[6] Iwase T, Uehara Y, Shinji H, Tajima A, Seo H, Takada K, et al *Staphylococcus epidermidis Esp* inhibits *Staphylococcus aureus* biofilm formation and nasal colonization. Nature. 2010;465: 346–349. 10.1038/nature09074 20485435

[7] Zipperer A, Konnerth MC, Laux C, Berscheid A, Janek D, Weidenmaier C, et al Human commensals producing a novel antibiotic impair pathogen colonization. Nature. 2016;535: 511–516. 10.1038/nature18634 27466123

[8] Chambers HF, DeLeo FR. Waves of resistance: *Staphylococcus aureus* in the antibiotc era. Nat Rev Microbiol. 2009;7: 629–641. 10.1038/nrmicro2200 19680247 PMC2871281

[9] European Food Safety Authority. Analysis of the baseline survey on the prevalence of methicillin-resistant *Staphylococcus aureus* (MRSA) in holdings with breeding pigs, in the EU, 2008—Part A: MRSA prevalence estimates. EFSA J. 2009;7: 1376–1458. 10.2903/j.efsa.2010.1597 PMC13085198 42006962

[10] Cuny C, Nathaus R, Layer F, Strommenger B, Altmann D, Witte W. Nasal colonization of humans with methicillin-resistant *Staphylococcus aureus* (MRSA) CC398 with and without exposure to pigs. PLoS One. 2009;4: e6800 10.1371/journal.pone.0006800 19710922 PMC2728842

[11] Garcia-Graells C, van Cleef BAGL, Larsen J, Denis O, Skov R, Voss A. Dynamic of livestock-associated methicillin-resistant *Staphylococcus aureus* CC398 in pig farm households: a pilot study. PLoS One. 2013;8: e65512 10.1371/journal.pone.0065512 23741497 PMC3669288

[12] Becker K, Schaumburg F, Fegeler C, Friedrich AW, Köck R, Prevalence of Multiresistant Microorganisms PMM Study. *Staphylococcus aureus* from the German general population is highly diverse. Int J Med Microbiol. 2017;307; 21–27. 10.1016/j.ijmm.2016.11.007 28017539

[13] Larsen J, Petersen A, Sørum M, Stegger M, van Alphen L, Valentiner-Branth P, et al Meticillin-resistant *Staphylococcus aureus* CC398 is an increasing cause of disease in people with no livestock contact in Denmark, 1999 to 2011. Euro Surveill. 2015;20: 30021. 10.2807/1560-7917.ES.2015.20.37.30021 PMC4902279 26535590

[14] Köck R, Loth B, Köksal M, Schulte-Wülwer J, Harlizius J, Friedrich AW. Persistence of nasal colonization with livestock-associated methicillin-resistant *Staphylococcus aureus* in pig farmers after holidays from pig exposure. Appl Environ Microbiol. 2012;78: 4046–7. 10.1128/AEM.00212-12 22447613 PMC3346418

[15] van Cleef BA, Monnet DL, Voss A, Krziwanek K, Allerberger F, Struelens M, et al Livestock-associated methicillin-resistant *Staphylococcus aureus* in humans, Europe. Emerg Infect Dis. 2011;17: 502–505. 10.3201/eid1703.101036 21392444 PMC3166010

[16] Larsen J, Petersen A, Larsen AR, Sieber RN, Stegger M, Koch A, et al Emergence of livestock-associated methicillin-resistant *Staphylococcus aureus* bloodstream infections in Denmark. Clin Infect Dis. 2017;65: 1072–1076. 10.1093/cid/cix504 28575216 PMC5850567

[17] George T, Lorenz MB, van Alen S, Hübner NO, Becker K, Köck R. MRSA colonization and infection among persons with occupational livestock exposure in Europe: prevalence, preventive options and evidence. Vet Microbiol. 2017;200: 6–12. 10.1016/j.vetmic.2015.10.027 26658156

[18] Angen Ø, Feld L, Larsen J, Rostgaard K, Skov R, Madsen AM, et al Transmission of MRSA to human volunteers visiting a swine farm. Appl Environ Microbiol. 2017;83: e01489–17. 10.1128/AEM.01489-17 28970219 PMC5691421

[19] Danish Integrated Antimicrobial Resistance Monitoring and Research Programme. DANMAP 2014—Use of antimicrobial agents and occurrence of antimicrobial resistance in bacteria from food animals, food and humans in Denmark. 2015 http://www.danmap.org/.

[20] Islam MZ, Espinosa-Gongora C, Damborg P, Sieber RN, Munk R, Husted L, et al Horses in Denmark are a reservoir of diverse clones of methicillin-resistant and -susceptible *Staphylococcus aureus*. Front Microbiol. 2017;8, 543 10.3389/fmicb.2017.00543 28421046 PMC5376617

[21] Ring HC, Thorsen J, Saunte DM, Lilje B, Bay L, Riis PT, et al The follicular skin microbiome in patients with hidradenitis suppurativa and healthy controls. JAMA Dermatology. 2017;153: 897–905. 10.1001/jamadermatol.2017.0904 28538949 PMC5710430

[22] Comeau AM, Douglas GM, Langille MGI. Microbiome Helper: a custom and streamlined workflow for microbiome research. mSystems. 2017;2: e00127–16. 10.1128/mSystems.00127-16 PMC5209531 28066818

[23] Rognes T, Flouri T, Nichols B, Quince C, Mahé F. VSEARCH: a versatile open source tool for metagenomics. PeerJ. 2016;4: e2584 10.7717/peerj.2584 27781170 PMC5075697

[24] Caporaso JG, Kuczynski J, Stombaugh J, Bittinger K, Bushman FD, Costello EK, et al QIIME allows analysis of high-throughput community sequencing data. Nat Methods. 2010;7: 335–336. 10.1038/nmeth.f.303 20383131 PMC3156573

[25] Kopylova E, Noé L, Touzet H. SortMeRNA: fast and accurate filtering of ribosomal RNAs in metatranscriptomic data. Bioinformatics. 2012;28: 3211–3217. 10.1093/bioinformatics/bts611 23071270

[26] Mercier C, Boyer F, Bonin A, Coissac E. SUMATRA and SUMACLUST: fast and exact comparison and clustering of sequences. 2013 http://metabarcoding.org/sumatra/.

[27] Dixon P. VEGAN, a package of R functions for community ecology. J. Veg. Sci. 2003;14: 927–930.

[28] R core Team. R: a language and environment for statistical computing. R Foundation for Statistical Computing, Vienna, Austria 2017 https://www.r-project.org/.

[29] Warnes GR, Bolker B, Bonebakker L, Gentleman R, Huber W, Liaw A, et.al gplots: various R programming tools for plotting data. 2019 https://cran.r-project.org/web/packages/gplots/.

[30] Dall’Antonia M, Coen PG, Wilks M, Whiley A, Millar M. Competition between methicillin-sensitive and -resistant *Staphylococcus aureus* in the anterior nares. J Hosp Infect. 2005;61: 62–7. 10.1016/j.jhin.2005.01.008 15893854

[31] Huang SS, Datta R, Rifas-Shiman S, Kleinman K, Placzek H, Lankiewicz JD, et al Colonization with antibiotic-susceptible strains protects against methicillin-resistant *Staphylococcus aureus* but not vancomycin-resistant enterococci acquisition: a nested case-control study. Crit Care. 2011;15: R210 10.1186/cc10445 21914221 PMC3334754

[32] Datta R, Quan V, Kim D, Peterson EM, Reynolds C, Meyers H, et al Protective effect of methicillin-susceptible *Staphylococcus aureus* carriage against methicillin-resistant *S*. *aureus* acquisition in nursing homes: a prospective cross-sectional study. Infect Control Hosp Epidemiol. 2014;35: 1257–1262. 10.1086/678062 25203179

[33] Ghasemzadeh-Moghaddam H, Neela V, van Wamel W, Hamat RA, Shamsudin MN, et al Nasal carriers are more likely to acquire exogenous *Staphylococcus aureus* strains than non-carriers. Clin Microbiol Infect. 2015;21: 998.e1–998.e7. 10.1016/j.cmi.2015.07.006 26183299

[34] Weese JS, Slifierz M, Jalali M, Friendship R. Evaluation of the nasal microbiota in slaughter-age pigs and the impact on nasal methicillin-resistant *Staphylococcus aureus* (MRSA) carriage. BMC Vet Res. 2014;10: 69 10.1186/1746-6148-10-69 24628871 PMC3995533

[35] Slifierz MJ, Friendship RM, Weese JS. Longitudinal study of the early-life fecal and nasal microbiotas of the domestic pig. BMC Microbiol. 2015;15: 184 10.1186/s12866-015-0512-7 26391877 PMC4578254

[36] Correa-Fiz F, Fraile L, Aragon V. Piglet nasal microbiota at weaning may influence the development of Glässer’s disease during the rearing period. BMC Genomics. 2016;17: 404 10.1186/s12864-016-2700-8 27230662 PMC4881051

[37] Espinosa-Gongora C, Larsen N, Schønning K, Fredholm M, Guardabassi L, Pieper D. Differential analysis of the nasal microbiome of pig carriers or non-carriers of *Staphylococcus aureus*. PLoS One. 2016;11: e0160331 10.1371/journal.pone.0160331 27509169 PMC4980049

[38] Kraemer JG, Ramette A, Aebi S, Oppliger A, Hilty M. Influence of pig farming on the human’s nasal microbiota: the key role of the airborne microbial communities. Appl Environ Microbiol. 2018;84: e02470–17. 10.1128/AEM.02470-17 29330190 PMC5835734

[39] Kraemer JG, Aebi S, Oppliger A, Hilty M. The indoor-air microbiota of pig farms drives the composition of the pig farmers’ nasal microbiota in a season-dependent and farm-specific manner. Appl Environ Microbiol. 2019;85: e03038–18. 10.1128/AEM.03038-18 30824439 PMC6495764

[40] Huttenhower C, Gevers D, Knight R, Abubucker S, Badger JH, Chinwalla AT, et al Structure, function and diversity of the healthy human microbiome. Nature. 2012;486: 207–214. 10.1038/nature11234 22699609 PMC3564958

[41] Bassis CM, Tang AL, Young VB, Pynnonen MA. The nasal cavity microbiota of healthy adults. Microbiome. 2014;2: 27 10.1186/2049-2618-2-27 25143824 PMC4138944

[42] De Boeck I, Wittouck S, Wuyts S, Oerlemans EFM, van den Broek MFL, Vandenheuvel D, et al Comparing the healthy nose and nasopharynx microbiota reveals continuity as well as niche-specificity. Front Microbiol. 2017;8: 2372 10.3389/fmicb.2017.02372 29238339 PMC5712567

[43] Costello EK, Lauber CL, Hamady M, Fierer N, Gordon JI, Knight R. Bacterial community variation in human body habitats across space and time. Science. 2009;326: 1694–1697. 10.1126/science.1177486 19892944 PMC3602444

[44] Kaspar U, Kriegeskorte A, Schubert T, Peters G, Rudack C, Pieper DH, et al The culturome of the human nose habitats reveals individual bacterial fingerprint patterns. Environ Microbiol. 2016;18: 2130–2142. 10.1111/1462-2920.12891 25923378

[45] Johnson JS, Spakowicz DJ, Hong BY, Petersen LM, Demkowicz P, Chen L, et al Evaluation of 16S rRNA gene sequencing for species and strain-level microbiome analysis. Nat Commun. 2019;10: 5029 10.1038/s41467-019-13036-1 31695033 PMC6834636

