# Supplementary figures and images for: Investigation of the human nasal microbiome in persons with long- and short-term exposure to methicillin-resistant Staphylococcus aureus and other bacteria from the pig farm environment
Source: PLoS One. 2020 Apr 30;15(4):e0232456. doi: 10.1371/journal.pone.0232456 (PMC7192431; doi:10.1371/journal.pone.0232456)

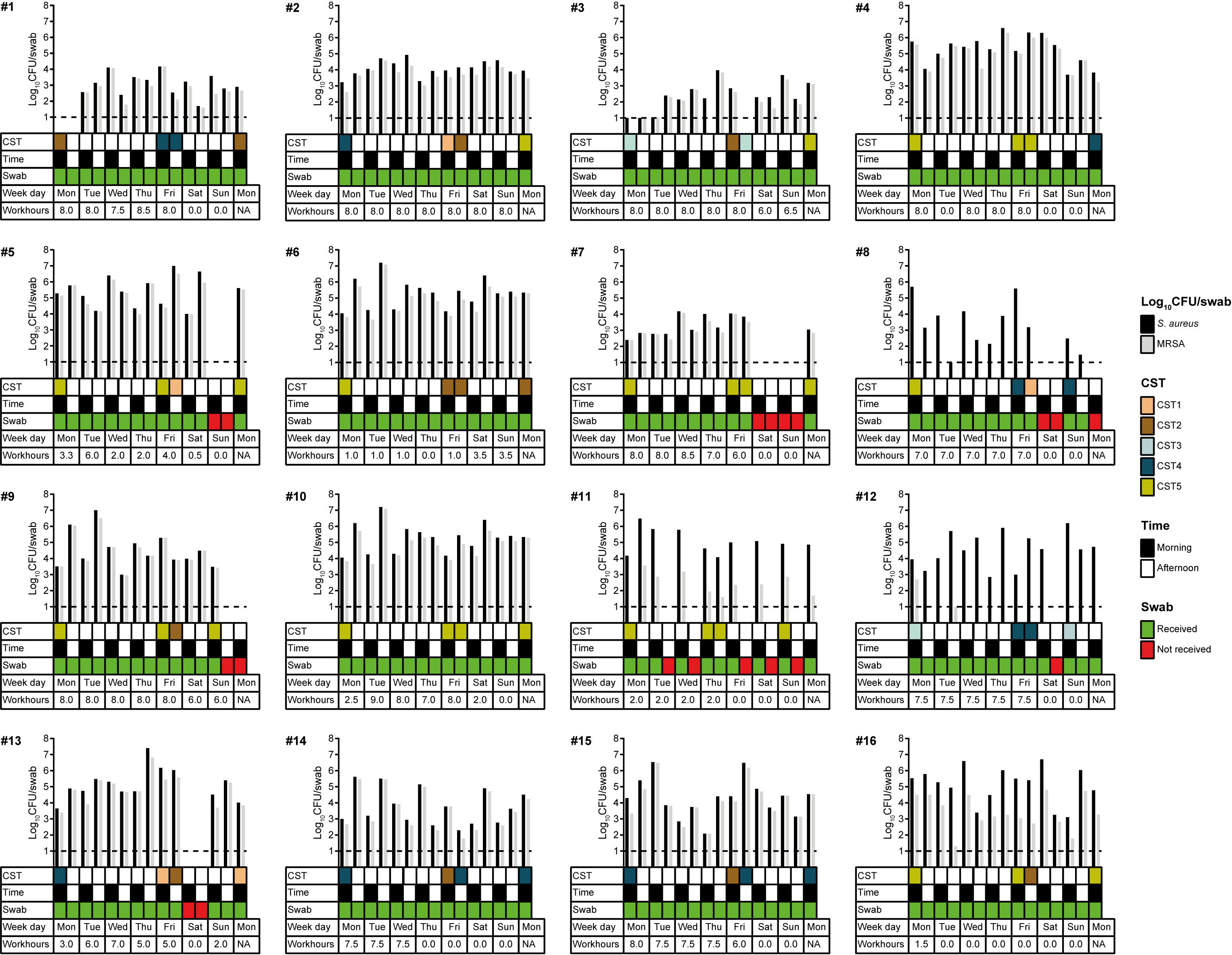

Supplement: S1 Fig — Log10-transformed CFU counts of Staphylococcus aureus and methicillin-resistant S. aureus and distribution of community state types (CSTs) among pig farm workers are shown. The dashed line indicates the limit of CFU detection. (TIF) [file pone.0232456.s001.tif]
